# Transactions of the Pathological Society of Philadelphia

**Published:** 1840-08-29

**Authors:** 


### MEDICAL EXAMINER.

DEVOTED TO MEDICINE, SURGERY, AND THE COLLATERAL SCIENCES.

No. 35.] PHILADELPHIA, SATURDAY, AUGUST 29, 1840. [Vol. III.

### TRANSACTIONS OF THE PATHOLOGICAL SOCIETY OF PHILADELPHIA.

*June*, 1840.

*Entozoaria.—Strongylus.*

Dr. Horner exhibited a collection of these worms, presented to him by Dr. Jordan, of Person, North Carolina, which had been discharged from the urethra of a man in that State. They amount to twelve or fourteen in number, and are about ten or twelve lines long, and one line in diameter. They are discharged periodically, and, as in similar cases, much suffering precedes this event.

Dr. Horner also exhibited a permanent tooth, the first bicuspate, which had been drawn out by its following the extraction of the first deciduous molar tooth of the upper jaw of a child. The accident appears to have been occasioned by the body of the bicuspate being wedged between the three fangs of the molar, and by its capsule adhering to the periosteum of that tooth. The specimen had been presented to him by Dr. Lucas Gee, of Mississippi.

